# Clarifying the Gateway Hypothesis and Conflict of Interest in Cannabis Research

**DOI:** 10.1002/npr2.70056

**Published:** 2025-09-09

**Authors:** Kenjiro Shiraishi

**Affiliations:** ^1^ Tanashi Kitaguchi Acupuncture and Moxa Clinic Tokyo Japan

## Abstract

This image visualizes the weighing of commercial incentives against research integrity, highlighting the challenge of maintaining transparency in CBD‐related studies.

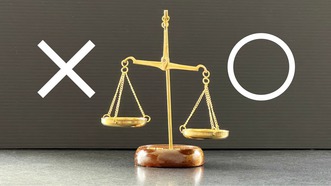

Dear Editor,

I read with great interest the recent article by Masataka et al., “Revisiting the Gateway Drug Hypothesis for Cannabis,” published in *Neuropsychopharmacology Reports* [1]. This study addresses an important topic, examining associations between cannabis use and subsequent use of other illicit substances among young people in Japan. I commend the authors for engaging with such a socially and medically significant issue.

I would, however, like to respectfully raise two concerns for further clarification and scholarly discussion.

Throughout the paper, the “gateway hypothesis” is referenced multiple times. However, a precise operational definition of this concept, along with a clear discussion of how correlation is distinguished from causation, appears limited. The notion that cannabis use leads to subsequent use of other illicit drugs is both highly sensitive and empirically contested.

For instance, Hall and Lynskey [2] reviewed the literature and emphasized that most studies supporting the gateway hypothesis are observational and cross‐sectional, making it difficult to infer causality from statistical associations alone. They highlighted the importance of distinguishing between temporal ordering and causal mechanisms, noting that social context and individual predisposition play crucial roles in shaping drug use trajectories.

Vanyukov et al. [3] further argued that the term “gateway” lacks theoretical precision and is often applied without clear operational criteria. Instead, they propose a “common liability to addiction” model, which posits that shared genetic, behavioral, and environmental risk factors better explain the observed progression of substance use than any linear gateway mechanism.

In addition, Degenhardt et al. [4], analyzing cross‐national data from the WHO World Mental Health Surveys, found that while the sequence of drug use initiation often follows a consistent pattern, this may reflect social availability or contextual factors rather than a causal effect of cannabis. They concluded that efforts to prevent cannabis use alone may not significantly reduce later drug use unless broader social determinants are also addressed.

Taken together, these studies suggest that the gateway hypothesis is conceptually ambiguous and empirically complex. A more explicit theoretical framing and acknowledgment of the limitations of causal inference would strengthen the clarity and interpretability of this important work.

The article in question declares, “Conflicts of Interest: None.” However, it appears that at least two of the authors are core members of GREEN ZONE JAPAN, an organization that publicly lists donations from companies selling cannabidiol (CBD) products on its official website. Furthermore, the study received partial funding from the Japanese Clinical Association of Cannabinoids, an organization that actively promotes the clinical use of cannabinoids. While such affiliations do not inherently imply misconduct or undue influence, the nature of the topic—cannabis and CBD, which carry significant social and commercial relevance—warrants comprehensive and transparent disclosure of potential conflicts of interest in accordance with international standards.

For example, the recommendations of the International Committee of Medical Journal Editors (ICMJE) state that authors must disclose all relationships, both financial and non‐financial, that could be perceived by others as potentially influencing their work [5]. Major medical journals such as *The BMJ* go further, declining to consider research funded by the tobacco industry for publication or peer review due to significant public health concerns [6]. Although the regulatory and scientific evaluation of the CBD and e‐cigarette industries remains underdeveloped in comparison, these fields similarly merit caution to preserve the integrity and neutrality of published research.

Indeed, recent studies have documented widespread industry involvement in CBD‐related research. Deary et al. [7] found that approximately 30% of CBD‐related publications involved authors with declared conflicts of interest, while many more included relationships that were not disclosed. This raises legitimate concerns about how such affiliations might influence study design, interpretation, and reporting.

Further illustrating the stakes, the U.S. National Institutes of Health (NIH) in 2018 terminated a major clinical trial (MACH15), which aimed to investigate the cardiovascular effects of “moderate” alcohol consumption, due to inappropriate involvement of the alcohol industry in both funding and study planning [8]. This case underscores the potential risks of industry interference in public health research.

Similarly, in the dietary supplement industry, concerns have been raised about the promotion of commercial products based on studies with undisclosed or poorly disclosed conflicts of interest. As Paul Offit noted in *Do You Believe in Magic?*, the supplement industry often operates under a regulatory structure that allows for aggressive marketing claims and industry‐sponsored research with insufficient oversight [9].

Together, these examples highlight the critical importance of rigorous conflict of interest disclosure and independent oversight, particularly in domains like CBD, alcohol, and dietary supplements—areas that may appear benign but are in fact characterized by substantial commercial influence and relatively lax regulation. As substances functioning both as consumer products and supplements, cannabis and CBD are susceptible to the same kinds of commercial pressures as alcohol and tobacco. For the sake of maintaining scientific credibility and public trust, it is imperative that ethical standards be strictly applied in such research.

Sincerely,

## Consent

The author has nothing to report.

## Conflicts of Interest

The author declares no conflicts of interest.

## Data Availability

The author has nothing to report.
